# 1-Aminocyclopropane-1-carboxylic acid induces resource reallocation in *Pyropia yezoensis* sporophytes

**DOI:** 10.3389/fpls.2025.1632530

**Published:** 2025-08-15

**Authors:** Toshiki Uji, Shun Sasaki, Hiroyuki Mizuta

**Affiliations:** Division of Marine Life Science, Faculty of Fisheries Sciences, Hokkaido University, Hakodate, Japan

**Keywords:** *Pyropia*, red algae, 1-aminocyclopropane-1-carboxylic acid, ethylene, plant hormone

## Abstract

Although the role of phytohormones in higher plants is well established, their role in macroalgae remains poorly understood. 1-Aminocyclopropane-1-carboxylic acid (ACC) is the immediate precursor of the plant hormone ethylene. However, recent studies suggested that ACC also acts as a signaling molecule to regulate growth and development independently of ethylene biosynthesis in land plants and red algae. This study investigated the role of ACC in the sporophytes of the red alga *Pyropia yezoensis*.

ACC treatment significantly inhibited the growth of *P. yezoensis* sporophytes, whereas ethephon, an ethylene-releasing compound, had no such effect. In addition, ACC treatment promoted the degradation of photosynthetic pigments, including chlorophyll, phycobiliprotein, and carotenoids. The investigation employed RNA sequencing to identify differentially expressed genes in response to ACC treatment in sporophytes. Notably, upregulated genes such as proteases were associated with catabolic processes. By contrast, genes related to anabolic processes such as photosynthesis, including light-harvesting complex protein and Calvin–Benson cycle enzymes, were downregulated in response to ACC treatment. ACC induced catabolic processes and repressed anabolic processes, indicating the promotion of resource reallocation in microscopic sporophytes.

## Introduction

Phytohormones regulate various developmental and physiological processes in plants, including traits of agronomic importance such as plant growth, development, stress tolerance, and nutrient availability (Wilkinson et al., 2012; Fenn and Giovannoni, 2021; Jia et al., 2022). To date, knowledge on phytohormone biosynthesis and signaling pathways has allowed the use of biotechnological tools to increase the yield or improve the quality of agricultural crops (Csukasi et al., 2009). In contrast to higher plants, the lack of knowledge about the role of phytohormones in macroalgae could hinder efforts to improve algal performance and yield.

Ethylene, a gaseous phytohormone, regulates various developmental processes such as seed germination, fruit ripening, and senescence, as well as responses to biotic and abiotic stresses (Bleecker and Kende, 2000; Koyama, 2014; Binder, 2020). Ethylene biosynthesis starts with the conversion of methionine to S-adenosyl l-methionine (SAM) by SAM synthetase, and SAM is converted to the non-protein amino acid 1-aminocyclopropane-1-carboxylic acid (ACC) by the enzyme ACC synthase (Adams and Yang, 1979). ACC is then converted to ethylene by ACC oxidase (Kende, 1993). Exogenous ACC has been employed as a proxy for ethylene in plant experiments. However, an increasing number of studies indicate that ACC acts as a signaling molecule beyond its function as an ethylene precursor (Van de Poel, 2020). In land plants, ACC appears to participate in the regulation of stress responses, cell expansion, cell wall function, stomatal development, and fertilization-related events (Tsang et al., 2011; Polko and Kieber, 2019; Vanderstraeten et al., 2019; Li et al., 2020; Katayose et al., 2021).

The multicellular red algal class Bangiophyceae, which contains *Porphyra* and *Pyropia*, is among the most important marine aquaculture crops commonly used to wrap sushi and onigiri. The life cycle of Bangiophyceae generally consists of the heterogeneous alternation of macroscopic gametophytes and microscopic sporophytes. Sporophytes are classified into conchocelis, which was considered a different species before the clarification of its life cycle (Drew, 1949; Kurogi, 1953; Iwasaki, 1961). In addition to land plants, there is accumulating evidence that ACC can serve as a signaling molecule independent of its role in ethylene signaling in the gametophytes of Bangiophyceae (Uji and Mizuta, 2022a). ACC induced gametogenesis and growth suppression in the gametophytes of the monoecious species *Pyropia yezoensis* (formerly *Neopyropia yezoensis*) and the dioecious species *Pyropia pseudolinearis* (Uji et al., 2016; Yanagisawa et al., 2019). Exogenous ACC analogs such as ACBC also promoted sexual reproduction in the same manner as ACC, whereas ethephon, an ethylene-releasing compound, did not stimulate sexual reproduction in *P. yezoensis* (Uji et al., 2020; Endo et al., 2021). In RNA sequencing (RNA-seq), transcripts associated with cell division, vesicular trafficking, and extracellular matrix (ECM) were upregulated in *Pyropia* gametophytes treated with ACC, whereas transcripts involved in translation, plastid transcription, and photosynthesis were downregulated (Uji et al., 2016; Uji et al., 2022b). Furthermore, phospholipase D and phosphatidic acid are required for signal transduction, ultimately leading to ACC-induced sexual reproduction in *P. yezoensis* (Uji et al., 2022a). In addition to sexual reproduction, the activation of ACC signaling can promote heat tolerance in *P. yezoensis* gametophytes by activating genes associated with antioxidant defense systems (Uji and Mizuta, 2022b).

Previous observations demonstrated morphological and structural differences between gametophytic and sporophytic generations in Bangiophyceae, including differences in morphology (blade vs. filamentous), chloroplast shape (stellate vs. parietal), growth behavior (diffuse growth vs. apical growth), and pit connection structure (absent vs. present) (Pueschel and Cole, 1985). Previous studies also revealed that the gametophytes and sporophytes of Bangiophyceae differ in the composition of ECM polysaccharides (Mukai et al., 1981) and inorganic carbon use (Wang et al., 2020). However, the differences in the roles of phytohormones between gametophyte and sporophyte generations remain poorly understood. Although several studies, as described above, have investigated the role of ACC in the gametophytes of *Pyropia*, information on its effects in the sporophyte stage is still limited. Therefore, the differences in ACC-related responses between the gametophyte and sporophyte generations are not yet fully clarified. To address this gap, we investigated the physiological and molecular responses of *P. yezoensis* sporophytes to ACC.

## Methods

### Algal materials and chemical treatments

The filamentous sporophytes of *P. yezoensis* strain TU-1 initiated from free-living conchocelis (U-51 1, C-O giant) (Kuwano et al., 1996) were cultured in sterile vitamin-free Provasoli’s enriched seawater (PES (Provasoli, 1968)) at 15°C under a 14-h-light/10-h-dark photoperiod using cool-white fluorescent lamps at 40 μmol photons m^−2^ s^−1^.

For the comparative experiment on the effects of ethephon and ACC, small tufts of vegetative sporophytes (approximately 0.2 to 0.3 mm in diameter) were cultured in 55-mm tissue culture dishes with 10 mL of medium containing 0 or 50 μM ACC (Tokyo Chemical Industry, Tokyo, Japan) or 50 μM ethephon (Fujifilm Wako Pure Chemical Corporation, Osaka, Japan) at 15°C under a 14-h-light/10-h-dark photoperiod using cool-white fluorescent lamps at 40 μmol photons m^−2^s^−1^. The concentration for ACC and ethephon treatments was determined according to our previous experiments with *P. yezoensis* gametophytes, in which this concentration effectively induced physiological responses while avoiding severe toxic effects. The culture medium was replaced once a week, and fresh medium containing the treatment reagents was added during each medium change. Growth was determined from the volume increase of the filamentous tufts as calculated from the mean diameters at the beginning and end of the experiments. As previously reported (Lin and Stekoll, 2007), the average diameter was the mean of two measurements taken at 90° of each other, and the tuft volume was estimated using the formula for the volume of a sphere: *V* = (1/6)π*D* ^3^ (*V* and *D* represent the volume and diameter of sporophyte tufts, respectively). The growth rate was calculated as the mean percent volume increase per day using the following formula: growth rate = [100(Vt − V0)/V0], where V0 is the initial tuft volume and Vt is the tuft volume at culture time.

### Evaluation of photochemical efficiency

After 2 weeks of ACC treatment, vegetative sporophytes were used for the measurement of the maximum photochemical efficiency of photosystem II (PS II) (*F*_v_/*F*_m_) to evaluate the effect of ACC on photosynthesis using a portable chlorophyll fluorometer (OS1p, Opti-Science, Inc., Hudson, NH, USA). Before measurement, the samples were dark-adapted for 15 min. Measurements were taken following the manufacturer’s protocol. Data are expressed as the mean ± standard deviation (SD) of five turfs for each condition.

### Quantification of pigment content

To examine the effect of ACC on the degradation of photosynthetic pigments [chlorophyll a (Chl *a*), phycoerythrin (PE), phycocyanin (PC), and carotenoids (Car)], sporophytes (fresh weight: ca. 0.02 g) were cultured in PES containing 0 or 50 μM ACC for 1 week at 15°C under a 14-h-light/10-h-dark photoperiod at 40 μmol photons m^−2^ s^−1^. The sporophytes were homogenized in 1 mL of 0.5 M phosphate buffer (pH 6.8) using a glass homogenizer. After centrifugation for 10 min at 12,000 ×*g* and 20°C, the supernatant was recovered to measure the PE and PC levels. Thereafter, 1 mL of 90% acetone was added to the homogenate, and the pellet was resuspended via vortexing. After centrifugation in the same manner, the Chl *a* and Car levels in the supernatant were determined. The supernatant was diluted five times with the same solvent used for extraction before light absorbance was measured using the U-1800 spectrophotometer (Hitachi, Tokyo, Japan). The PE and PC levels were calculated as described previously (Beer and Eshel, 1985), and the Chl *a* and Car contents were determined as previously reported (Seely et al., 1972). These experiments were repeated five times.

### RNA preparation

All sporophytes from each sample were frozen in liquid nitrogen and immediately stored at −80°C until RNA extraction. Total RNA from algal samples was extracted as described previously (Drew, 1949). The quantity and integrity of the RNA samples were assessed using the NanoDrop^™^ 2000 spectrophotometer (Thermo Fisher Scientific, Waltham, MA, USA), Qubit^™^ 4 fluorometer (Thermo Fisher Scientific), and Agilent 2100 bioanalyzer (Agilent Technologies, Santa Clara, CA, USA). Four libraries of complementary DNA (0 and 50 μM ACC, three replicates each) for *P. yezoensis* were constructed and subsequently sequenced using the Illumina NovaSeq 6000 instrument at Rhelixa Inc. (Tokyo, Japan).

### RNA-seq

Low-quality reads and adapter sequences were trimmed from the obtained reads using fastp (Chen et al., 2018). After trimming, STAR (Dobin et al., 2013) was used to map the high-quality reads to an in-house gene model of *P. yezoensis*, which had been constructed with a reference genome (ASM982973v1) and other in-house transcriptome data using the BRAKER 2.1.6 (Brůna et al., 2021) pipeline. Next, the normalized expression of each gene was calculated using RSEM (Li and Dewey, 2011) as transcripts per million. Differentially expressed genes (DEGs) between the 0 and 50 μM ACC groups were identified by edgeR (Robinson et al., 2010) using the following criteria: false discovery rate <0.05 and |log_2_ fold change| >1. In addition, RNA-Seq data previously performed in our laboratory (Uji et al., 2016) were used to compare DEGs between sporophytes and gametophytes following ACC treatment.

### Annotation and Gene Ontology enrichment analysis

To assess the biological significance of the DEGs, we conducted Gene Ontology and Kyoto Encyclopedia of Genes and Genomes (KEGG) pathway enrichment analyses. GO terms were assigned to all genes using eggNOG-mapper v2 online (http://eggnog-mapper.embl.de/) (Huerta-Cepas et al., 2019; Cantalapiedra et al., 2021) with the default parameters except that the min_hit_e-value was set to 0.05. The topGO (Alexa and Rahnenfuhrer, 2023) R package was used for the GO enrichment analysis, and GO terms with *P <*0.05 were considered significantly enriched in the DEGs.

In addition, we employed KOBAS 3.0 software (Bu et al., 2021) to conduct KEGG annotation and enrichment analysis based on the KEGG PATHWAY Database (https://www.genome.jp/kegg/pathway.html) (Kanehisa and Goto, 2000). Pathways with corrected *p*-value (*q*-value) <0.05 were defined as significantly enriched pathways for the DEGs.

### Quantitative PCR

Quantitative PCR (qPCR) analysis was performed as described previously (Uji et al., 2019). The mRNA levels were calculated using the 2^−ΔΔCt^ method and normalized to the level of the 18S ribosomal RNA (*Py18SrRNA*) gene (Uji et al., 2016). The relative expression was calculated as the ratio of the mRNA level to the transcription level of samples without ACC treatment. All experiments were performed in three biological replicates. Additional File 1: **Supplementary Table S1** presents the list of primers used in this study.

### Statistical analysis

Data are expressed as mean ± standard deviation and analyzed using the Mann–Whitney *U*-test. For all analyses, *P <*0.05 was considered statistically significant.

## Results and discussion

### Effect of ACC treatment on the growth of *P. yezoensis* sporophytes

**Figure 1:**
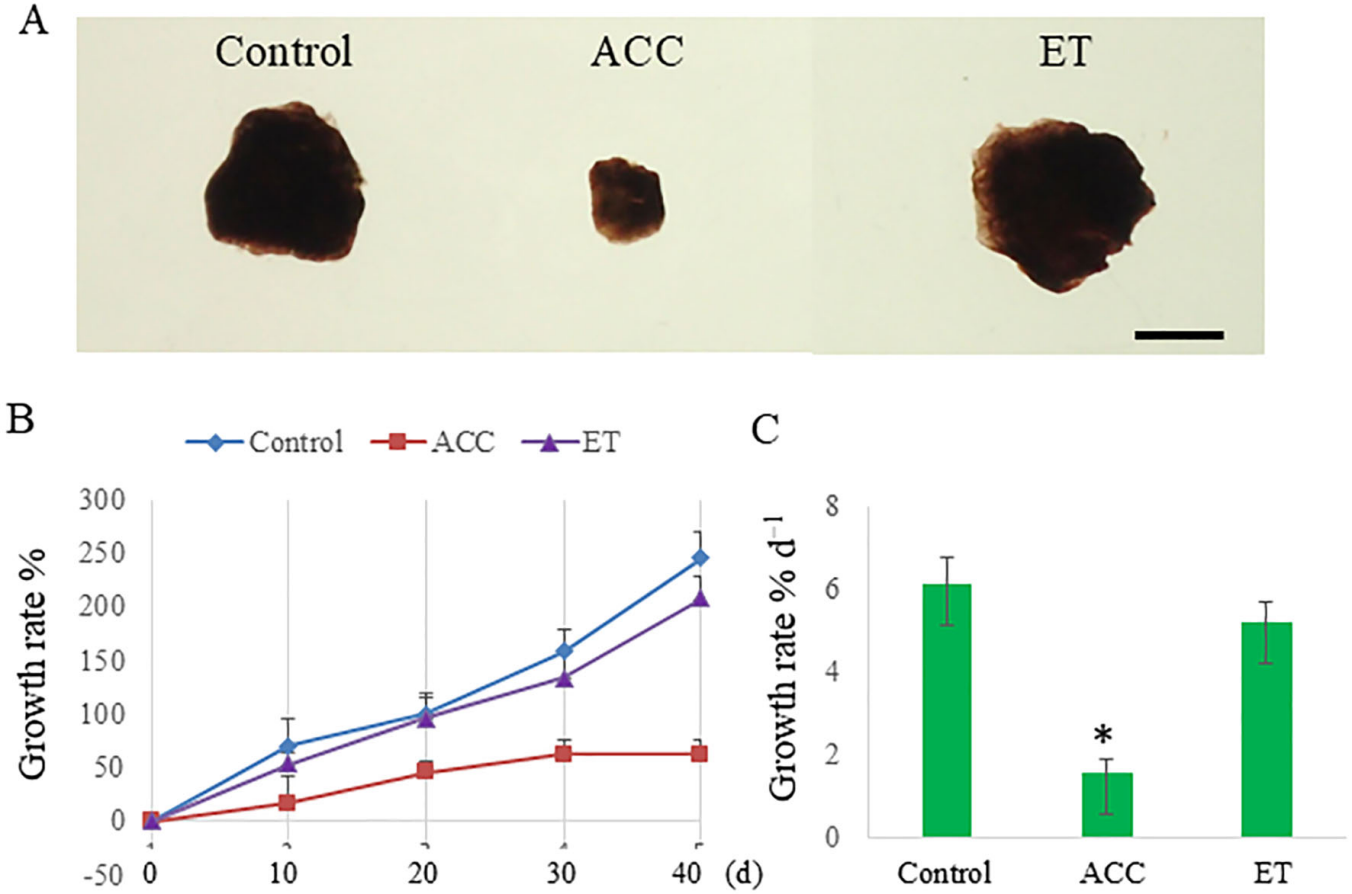
1-Aminocyclopropane-1-carboxylic acid (ACC)-induced growth inhibition in *Pyropia yezoensis* sporophytes. **(A)** Representative photographs of ACC-treated *P. yezoensis* sporophytes. Scale bar = 5 mm. **(B)** Sporophytes during 40 days of culture with 0 or 50 μM ACC or 50 μM ethephon (ET). **(C)** Growth rate of sporophytes cultured with 0 or 50 μM ACC or 50 μM ET. Data are expressed as the mean ± standard deviation of six tufts for each condition. The asterisks indicate significant differences at *P* < 0.05 between the control and treatment groups.

As a first step in clarifying the effects of ACC on *P. yezoensis* sporophytes, possibly independent of ethylene, we compared the effects of ACC and ethephon on growth and reproduction. When sporophytes were cultured for 40 days in a medium containing 50 μM ACC, the growth rate was 62.5%, whereas untreated (control) sporophytes exhibited a growth rate of 245.8% (**Figures 1A, B**). By contrast, ethephon did not significantly inhibit sporophyte growth (208.3%). The growth rate per day was 1.5% day^−1^ in ACC-treated sporophytes versus 6.1% day^−1^ in control sporophytes and 5.2% day^−1^ in ethephon-treated sporophytes (**Figure 1C**).

A previous study reported increases in ACC levels during the transition from the vegetative to the reproductive stages and showed that ACC promotes the transition from vegetative conchocelis to conchosporangia in *Pyropia haitanensis* (Niu et al., 2024). In our study, we did not observe similar effects of ACC on the maturation of *P. yezoensis* sporophytes under the conditions tested (data not shown). This discrepancy may reflect species-specific differences or variations in experimental conditions, including developmental stage, ACC concentration, light, and temperature. Further studies are needed to determine whether ACC plays a broader role in the life cycle regulation of different red algal species.

### Effect of ACC treatment on photosynthetic pigment content and photochemical efficiency in *P. yezoensis* sporophytes

**Figure 2:**
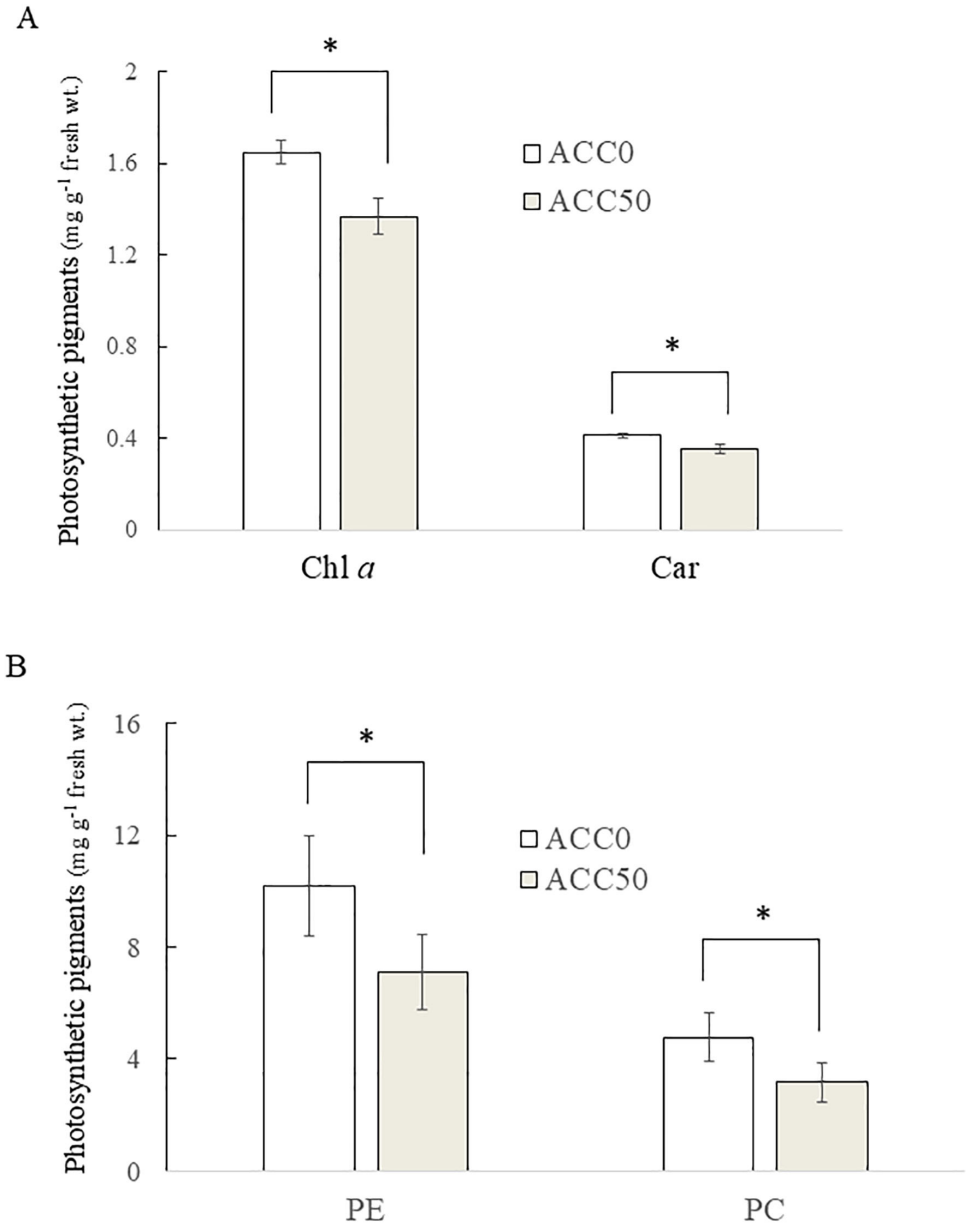
Effect of 1-aminocyclopropane-1-carboxylic acid (ACC) on photosynthetic pigment degradation in *Pyropia yezoensis* sporophytes. The contents of chlorophyll a (Chl *a*), carotenoids (Car) **(A)**, phycoerythrin (PE), and phycocyanin (PC) **(B)** were measured using sporophytes cultured in medium containing 0 or 50 μM ACC. Data are expressed as the mean ± standard deviation of five independent experiments (mg g^−1^ fresh wt.). The asterisks indicate significant differences at *P* < 0.05 between the control and treatment groups.

Our previous study illustrated that ACC promotes the degradation of photosynthetic pigments, namely, Chl *a*, PE, PC, and Car, in gametophytes (Uji et al., 2016). Thus, we examined the effects of ACC on photosynthetic pigment content in sporophytes. Treatment with 50 μM ACC resulted in 17.0% and 29.4% decreases in Chl *a* and PE levels, respectively, compared to the control findings (**Figure 2**). Statistical analysis revealed significant differences in Chl *a*, PE, PC, and Car contents between ACC-treated and untreated sporophytes.

**Figure 3:**
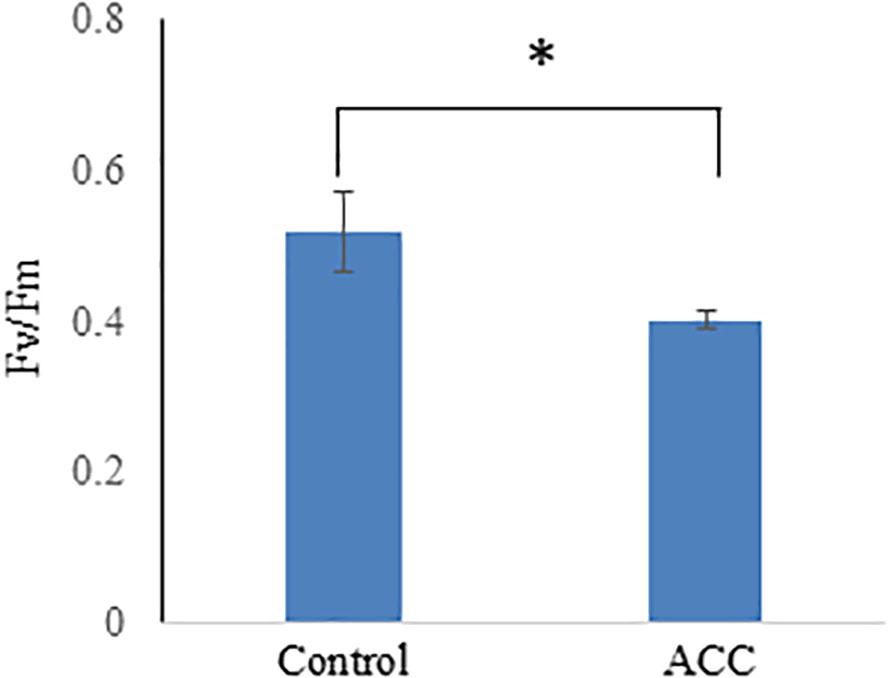
Effect of 1-aminocyclopropane-1-carboxylic acid (ACC) on the photosynthetic capacity in *Pyropia yezoensis* sporophytes. The maximum photochemical efficiency (*F*_v_/*F*_m_) was assessed and measured using sporophytes cultured in a medium containing 0 or 50 μM ACC. Data are expressed as the mean ± SD of five turfs for each condition. The asterisks indicate significant differences at *P* < 0.05 between the control and treatment groups.

Maximum quantum efficiency (*F*_v_/*F*_m_) using pulse amplitude modulation techniques was employed to assess the impact of ACC treatment on the photosynthetic capacity in *P. yezoensis*. The *F*_v_/*F*_m_ value of sporophytes treated without ACC was 0.518, whereas that of sporophytes supplemented with ACC was 0.402 (**Figure 3**). Such a decline in photosynthetic efficiency is consistent with the observed reduction in photosynthetic pigment content. In higher plants, leaf senescence is typically accompanied by decreased photosynthetic activity and chlorophyll degradation as part of nutrient remobilization processes during the final stages of development (Lim et al., 2007). Our findings suggest that ACC may induce senescence-like physiological changes in the sporophytes of *P. yezoensis*, leading to reduced photosynthetic performance similar to the senescence processes described in terrestrial plants.

### Transcriptomic responses to ACC treatment

To further clarify the role of ACC in *P. yezoensis* sporophytes, we compared RNA-seq data between ACC-treated and untreated sporophytes. ACC-treated sporophytes were sampled at 3 days after treatment. Raw data generated by sequencing ranged from 18.0 to 21.5 million reads per sample. After filtering, 17.3–21.2 million clean reads were obtained, and the mapping rate ranged 89.0%–91.2%. A summary of the obtained RNA-seq datasets and the mapping rate of clean reads is presented in **Table 1**.

**Table 1:** Summary of transcriptome analysis in *P. yezoensis* sporophytes.

Sample name	Raw reads	Clean reads	GC content (%)	Mapping rate (%)
ACC0-1	21,533,486	20,712,058	64.8	89.0
ACC0-2	19,369,066	18,668,264	64.5	90.7
ACC0-3	23,144,950	21,207,270	65.7	90.4
ACC50-1	20,094,898	19,326,914	64.9	90.5
ACC50-2	18,020,880	17,323,998	64.8	90.0
ACC50-3	21,587,030	19,956,114	66.0	91.2

**Figure 4:**
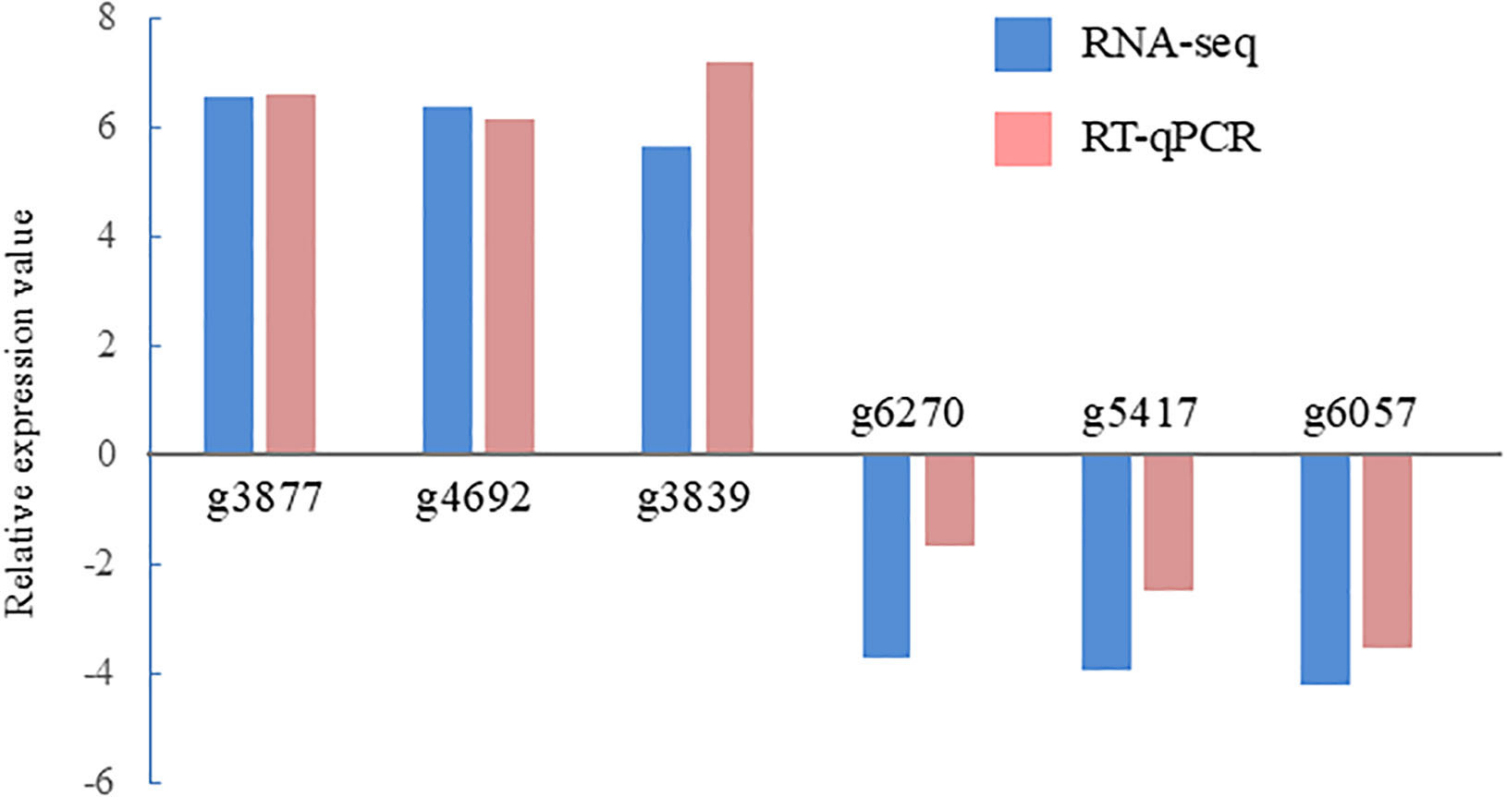
Validation of RNA sequencing (RNA-seq) data using reverse transcription-quantitative polymerase chain reaction (RT-qPCR). Six representative genes were selected to validate the RNA-seq data by RT-qPCR. The pink bars represent the mean log_2_-fold change obtained by RT-qPCR, and the blue bars represent the RNA-seq data. The results are presented as relative expression compared to that in untreated sporophytes. The primers used for RT-qPCR are listed in **Supplementary Table S1**.

In total, 438 DEGs were identified between the control and ACC treatment groups, including 167 upregulated and 271 downregulated genes in response to ACC (**Supplementary Tables S2**, **S3**). Six DEGs (three upregulated and three downregulated genes) were selected for qPCR to validate the accuracy of the RNA-seq data. As illustrated in **Figure 4**, the expression of the selected genes was similar in the reverse transcription-qPCR and RNA-seq data, indicating that the RNA-seq results were reliable. The representative genes found to be differentially expressed in response to ACC treatment in sporophytes are presented in **Tables 2**, **3**. The identification of these DEGs led us to perform further analyses, such as KEGG and GO enrichment, to explore their potential roles in metabolic processes and physiological changes induced by ACC, as discussed below.

**Table 2:** List of upregulated genes in *P. yezoensis* sporophytes treated with ACC.

Contig ID	Functional categories	Description	Fold change
g3877	Proteolysis	Trypsin-like protease	7.08
g3839	Proteolysis	Subtilisin-like protease	5.78
g6073	Proteolysis	Subtilisin-like protease	5.64
g5197	Proteolysis	Neprilysin-like protease	5.06
g8209	Proteolysis	Trypsin-like protease	5.01
g2889	Glycosidase	Glycoside hydrolase family	4.56
g8207	Proteolysis	Trypsin-like protease	4.49
g2380	Proteolysis	Subtilisin-like protease	4.28
g4037	Proteolysis	Subtilisin-like protease	4.27
g6204	Proteolysis	Subtilisin-like protease	3.86
g7623	Proteolysis	Trypsin-like protease	3.59
g3410	Glycosidase	Beta-glucosidase	3.16
g6232	Branched-chain amino acid catabolic process	Branched chain keto acid dehydrogenase E1 subunit alpha	2.37

**Table 3:** List of downregulated genes in *P. yezoensis* sporophytes treated with ACC.

Contig ID	Functional categories	Description	Fold change
g6057	Photosynthesis	Light-harvesting complex protein	2.99
g1883	Photosynthesis	Light-harvesting complex protein	2.57
g3912	Photosynthesis	Rieske FeS protein	2.17
g2936	Photosynthesis	Light-harvesting complex protein	1.99
g4048	Photosynthesis	Fructose-bisphosphate aldolase	1.88
g6242	Photosynthesis	Ribose-5-phosphate isomerase	1.63
g6251	Photosynthesis	Light-harvesting complex protein	1.51
g2482	Photosynthesis	Photosystem II repair protein	1.27
g2122	Photosynthesis	PSII 6.1-kDa protein	1.26

**Figure 5:**
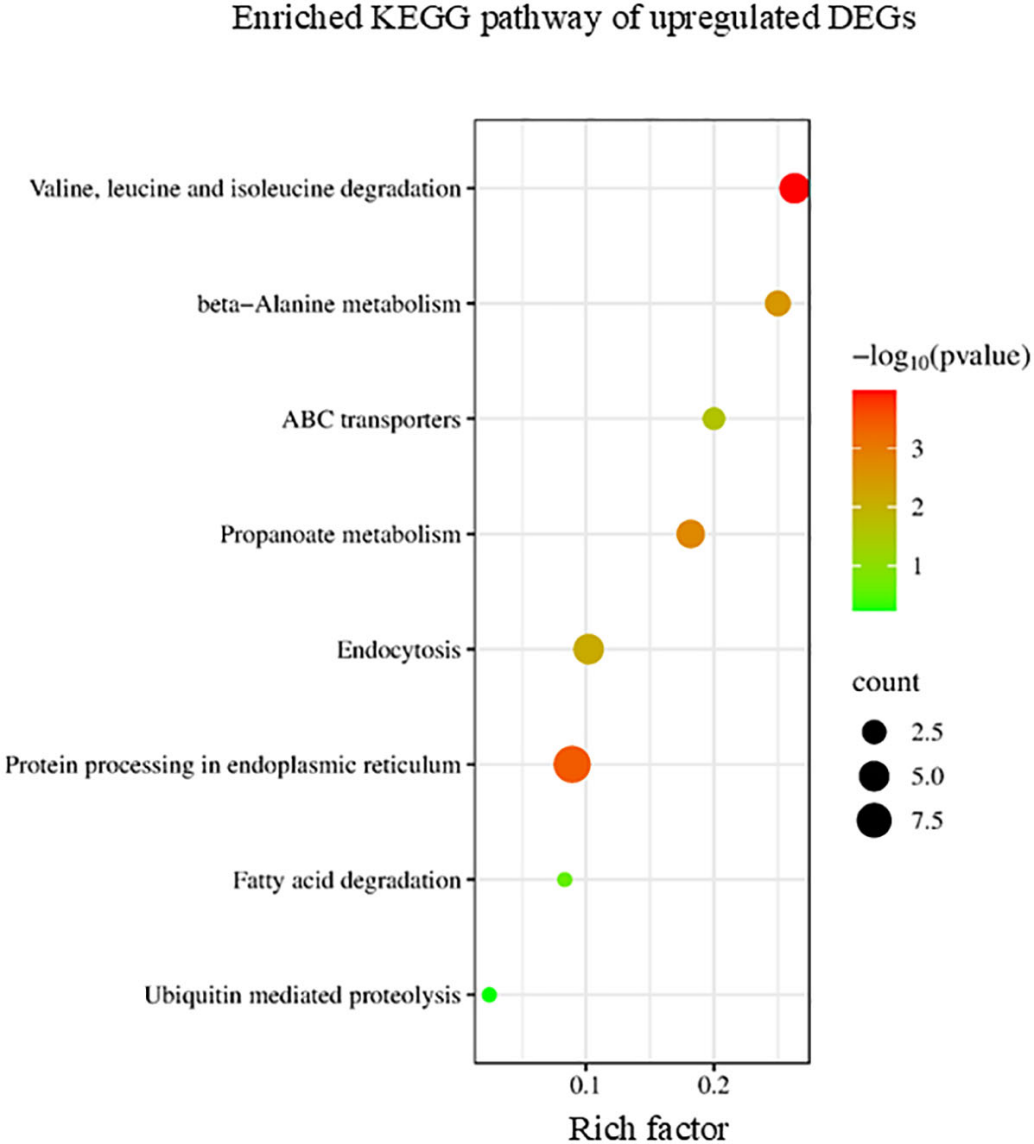
Kyoto Encyclopedia of Genes and Genomes (KEGG) enrichment analysis of the upregulated differentially expressed genes (DEGs). The degree of enrichment increased as the rich factor increased. The larger dots indicate higher numbers of differential genes enriched by the pathway.

KEGG enrichment analysis indicated that upregulated DEGs induced by ACC could be categorized into several pathways, including ubiquitin-mediated proteolysis, endocytosis, and valine, leucine, and isoleucine degradation (**Figure 5**). Protein degradation, which allows the recycling of nitrogen and other nutrients, is probably the most important degradation process that occurs during senescence (Roberts et al., 2012). In addition, the intracellular vesicle trafficking system transports cargo to specialized vacuolar compartments for digestive proteolysis mediated by various proteases (Wang and Schippers, 2019). Branched-chain amino acid (BCAA) degradation provides energy for plants during periods of extended darkness, early phases of germination, and late phases of senescence (Gipson et al., 2017).

**Figure 6:**
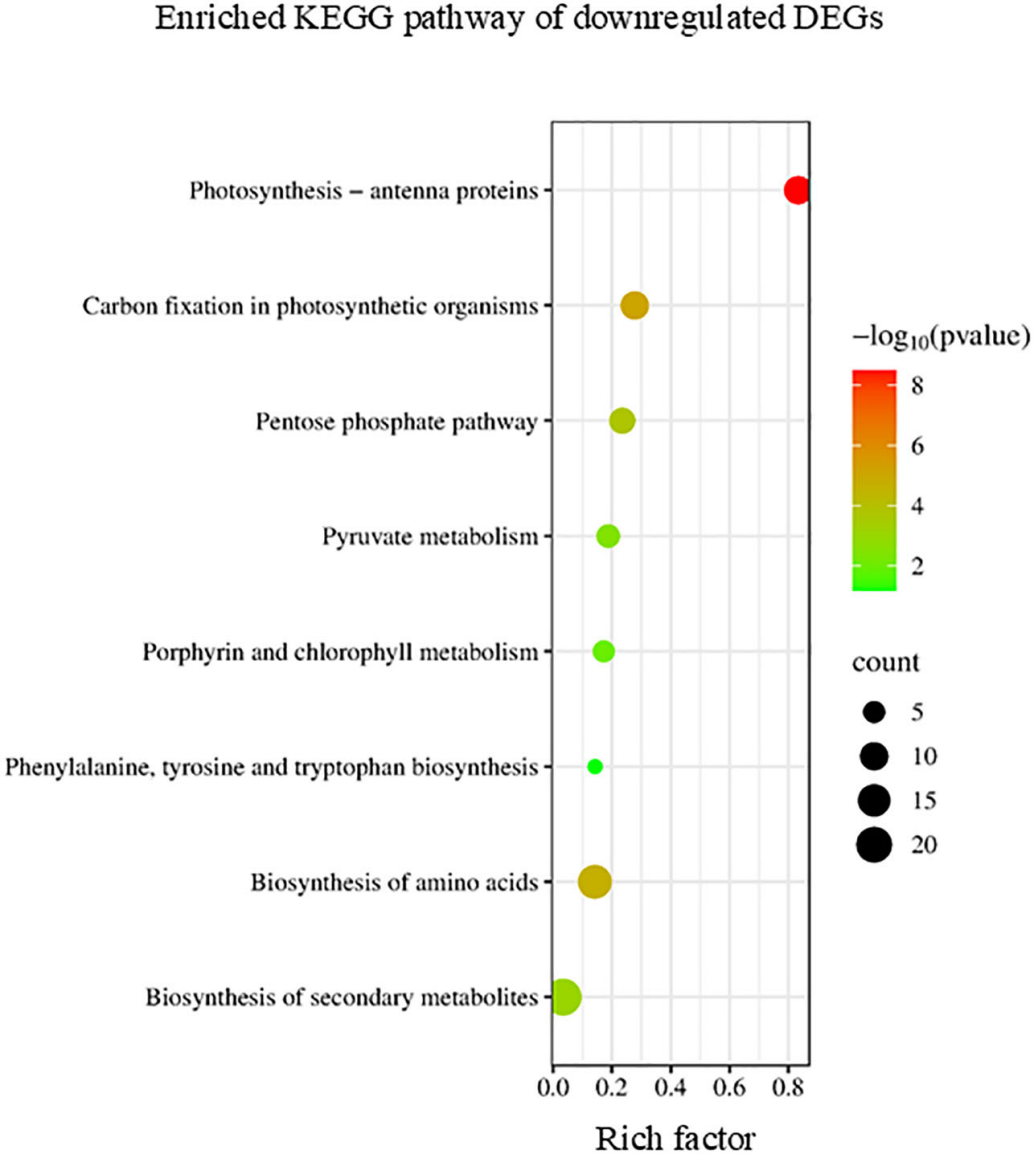
Kyoto Encyclopedia of Genes and Genomes (KEGG) enrichment analysis of the downregulated differentially expressed genes (DEGs). The degree of enrichment increased as the rich factor increased. The larger dots indicate higher numbers of differential genes enriched by the pathway.

Conversely, the KEGG enrichment analysis revealed that downregulated DEGs were categorized into pathways including photosynthesis, pentose phosphate pathway, and amino acid biosynthesis (**Figure 6**). In higher plants, leaf senescence is accompanied by extensive metabolic transformation from biosynthesis to degradation (Chaomurilege et al., 2023).

**Figure 7:**
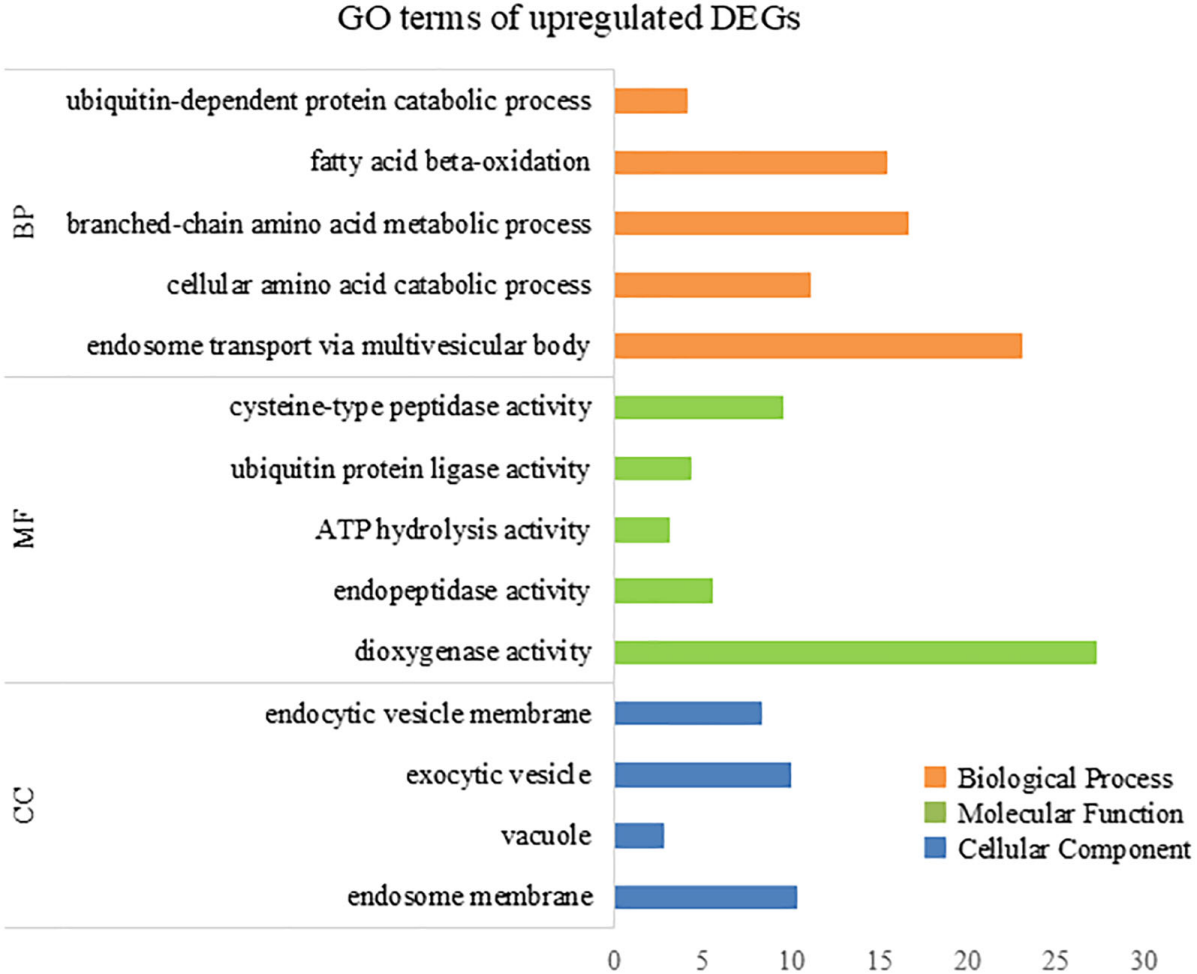
The numbers of enriched Gene Otology (GO) terms for the upregulated differentially expressed genes (DEGs). The GO terms are presented for three main categories: biological processes, molecular functions, and cellular components. Each GO term is listed in ascending order by *p*-value.

**Figure 8:**
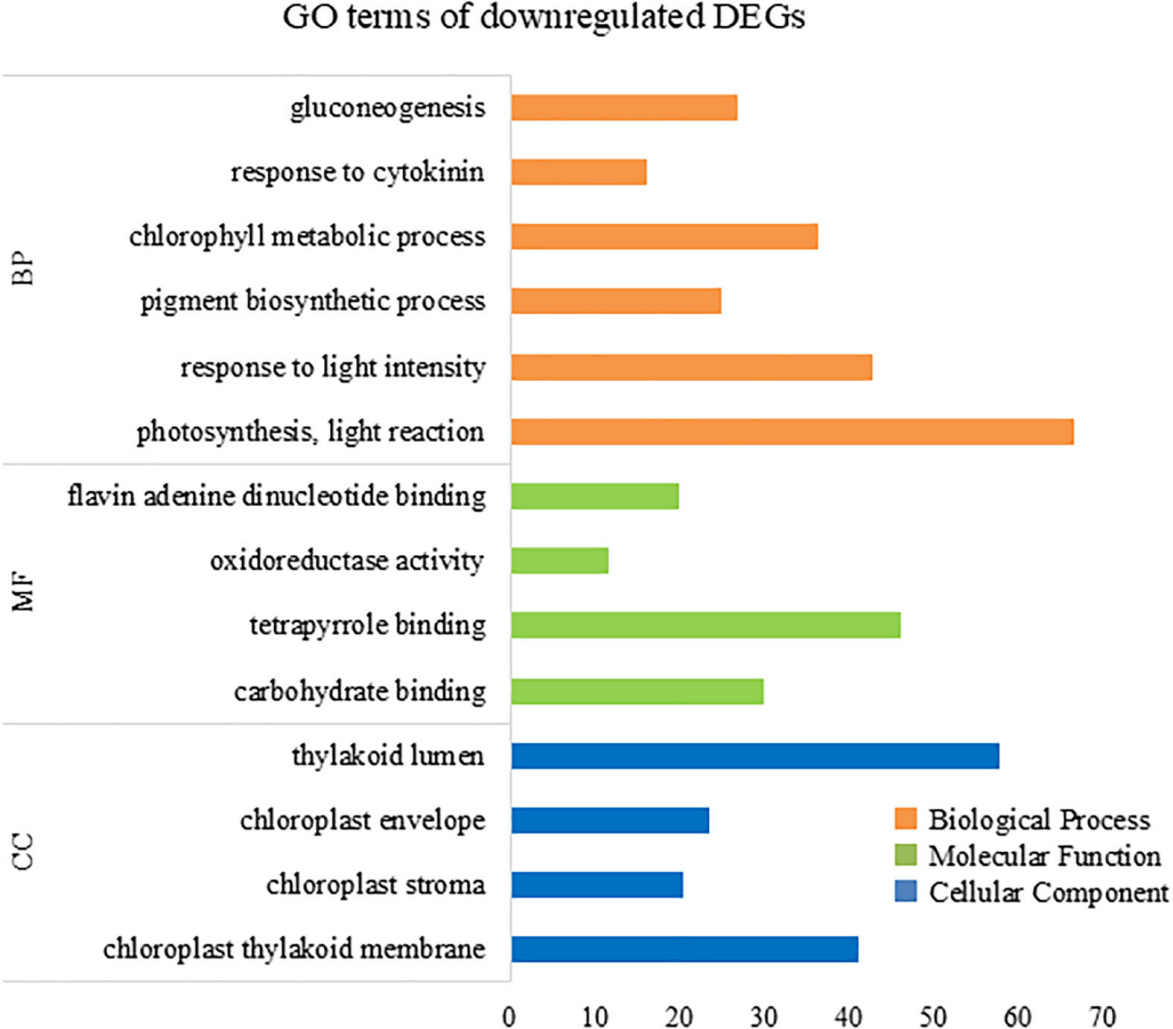
Numbers of enriched Gene Otology (GO) terms for the downregulated differentially expressed genes (DEGs). The GO terms are presented for three main categories: biological processes, molecular functions, and cellular components. Each GO term is listed in ascending order by *p*-value.

To further elucidate the biological processes, molecular functions, and cellular components associated with DEGs, we performed GO analysis using eggNOG-mapper and topGO. In the upregulated DEGs, GO terms related to cellular catabolic processes, proteasome-related pathways, and molecular and protein transport were enriched (**Figure 7**), indicating that ACC induced protein catabolic processes. In the downregulated DEGs, GO terms related to photosynthesis, the photosystem, and chloroplast components were enriched (**Figure 8**), in line with the result that ACC induced photosynthetic pigment degradation. Consequently, KEGG enrichment analysis and GO analysis revealed that DEGs in ACC-treated sporophytes include genes associated with plant senescence.

In the RNA-seq analysis, we identified 10 upregulated genes encoding proteases, including serine proteases and metalloproteases (**Table 2**; Additional File 1: **Supplementary Figure S1**). Among them, four genes encoded trypsin-like proteases, while five genes encoded subtilisin-like proteases. Subtilisin-like proteases belong to a large family found across diverse organisms and share conserved catalytic domains (Dodson and Wlodawer, 1998; Tripathi and Sowdhamini, 2006; Kim et al., 2016; Patel, 2017). Additionally, we identified one neprilysin-like metalloprotease gene (g5197) possessing typical conserved motifs, suggesting that it belongs to the M13 family of zinc metallopeptidases (Oefner et al., 2000; Turner et al., 2001; Bland et al., 2008; Yang et al., 2016). The upregulation of various proteases, including subtilisin-like and neprilysin-like proteases, in ACC-treated sporophytes suggests that ACC may enhance the protein degradation pathways. In higher plants, increased proteolytic activity during senescence facilitates nitrogen remobilization by breaking down proteins into amino acids, which can then be transported to developing tissues or storage organs (Lim et al., 2007). The observed induction of these proteases in *P. yezoensis* sporophytes may indicate a similar process, whereby ACC triggers catabolic pathways to recycle nitrogen and other nutrients during stress or senescence-like conditions. Further biochemical analyses will be necessary to confirm whether these transcriptomic changes translate into increased protease activity and contribute to nutrient remobilization in red algae.

In addition to proteases, the results demonstrated that the DEG (g6232) encoding the mitochondrial branched-chain alpha-ketoacid dehydrogenase complex (BCKDC) was upregulated. Among amino acids, the BCAAs leucine, isoleucine, and valine are proposed to provide their downstream catabolic products, namely, acetoacetate, acetyl-CoA, and propionyl-CoA, respectively, to the TCA cycle for energy generation (Dimou et al., 2022). In mammals, BCKDC consists of multiple copies of three proteins: keto acid dehydrogenase/carboxylase E1 (E1α and E1β), dihydrolipoyl acyltransferase E2, and dihydrolipoyl dehydrogenase E3. This complex, with a molecular weight of several megadaltons, catalyzes the second step of BCAA degradation by converting branched-chain ketoacids (intermediates in BCAA biosynthesis and catabolism) into acyl-CoA esters (Harris et al., 2001). Previous studies revealed that transcripts of BCAA catabolic genes increase during prolonged darkness, which is a well-known external stimulus capable of accelerating leaf senescence (Binder, 2010). During carbon deficiency, BCAA degradation products can serve as alternative energy sources by being fed into the TCA cycle in *Arabidopsis* (Angelovici et al., 2013). Thus, the induction of BCKDC from *P. yezoensis* suggests that ACC regulates BCAA catabolism, thereby contributing to algal fitness under energy-limited conditions.

Although chloroplasts contain their own genome, a large fraction of chloroplast proteins are encoded by the nuclear genome. Chloroplasts contain up to 70% of leaf proteins, the majority of which are photosynthetic proteins, including ribulose bisphosphate, carboxylase/oxygenase, and chlorophyll-binding/light-harvesting complex proteins (Fu et al., 2022). As presented in **Table 3**, transcriptome analysis illustrated that nuclear genome-encoded, chloroplast-targeted proteins such as light-harvesting complex proteins (g6057, g1883, and g2936) and Rieske FeS protein (g3912) were downregulated by ACC treatment in sporophytes. In addition to the photosynthetic apparatus, genes encoding enzymes involved in the Calvin–Benson cycle, including fructose-1,6-biphosphate aldolase (g4048), were downregulated in sporophytes treated with ACC.

Consistent with the downregulation of photosynthesis-associated genes, sporophytes treated with ACC exhibited photosynthetic pigment degradation (**Figure 2**). In addition to a role in light harvesting, PE in red algae plays a more important role in maintaining the nitrogen pool (Mizuta et al., 2002). In higher plants, Chl degradation contributes to nitrogen remobilization during leaf senescence (Hörtensteiner, 2006). Similarly, the degradation of photosynthetic pigments, especially PE, induced by the action of ACC might be involved in nitrogen remobilization in *P. yezoensis*.

Senescence is the last stage of plant development, and it is accompanied by a transition from nutrient assimilation to nutrient remobilization (Roberts et al., 2012). In plants, it is known that, during senescence, many major macromolecules, including proteins, lipids, and nucleic acids, are degraded in a process triggered by the reprogramming of thousands of genes (upregulation or downregulation) in response to specific senescence-promoting factors such as the plant hormone ethylene (Kim et al., 2016). In higher plants, senescence is primarily regulated by ethylene, which acts as a key signaling molecule. ACC serves as the immediate precursor of ethylene and does not directly induce senescence processes. In contrast, our findings suggest that in *P. yezoensis*, ACC itself may directly trigger gene reprogramming associated with senescence-like responses, possibly independent of ethylene production.

### Comparison of DEGs between sporophytes and gametophytes following ACC treatment

Our previous study provided the comprehensive transcriptome data of *P. yezoensis* gametophytes treated with ACC (Uji et al., 2016). Therefore, we compared DEGs in response to ACC exposure between gametophytic and sporophytic generations (**Table 4**). Endocytosis-related genes were upregulated in gametophytes and sporophytes. g7418, which encodes flotillin, was identified as a gene largely responsive to ACC in both gametophytes and sporophytes. Flotillins are membrane-associated proteins considered to function in a number of cellular contexts such as endocytosis, providing molecular scaffolding for membrane rafts that act by demarcating sites for the delivery of specific cargo (Otto and Nichols, 2011). Vacuolar protein sorting-associated protein 4 (Vps4) (g5362) and charged multivesicular body protein 5 (CHMP5) (g8621) associated with endosomal sorting complex required for transport III (ESCRT-III) (Otto and Nichols, 2011; Yang and Wang, 2022) were also upregulated in gametophytes and sporophytes treated with ACC. Nutrient starvation-induced endocytosis and degradation of various membrane proteins via the ESCRT-dependent pathway are crucial for maintaining critical amino acid levels (Alonso et al., 2016). In higher plants, the ESCRT-III subunit CHMP is required for the autophagic degradation of plastid proteins (Spitzer et al., 2015). Similarly, ACC can activate the ESCRT-dependent endosomal pathway to recycle degraded photosynthetic pigment proteins in *P. yezoensis*.

**Table 4:** Comparison of DEGs between the gametophytes (GA) and sporophytes (SP) treated with ACC.

Contig ID	Functional categories	Description	Fold change GA vs. SP
g7418	Vesicular trafficking	Flotillin-2	6.68 vs. 5.34
g5362	Vesicular trafficking	Vacuolar protein sorting-associated protein 4	4.92 vs. 1.57
g8621	Vesicular trafficking	Charged multivesicular body protein 5	3.99 vs. 1.08
g3839	Proteolysis	Subtilisin-like protease	—– vs. 5.78
g6073	Proteolysis	Subtilisin-like protease	—– vs. 5.64
g8207	Proteolysis	Trypsin-like protease	—– vs. 4.49
g8209	Proteolysis	Trypsin-like protease	—– vs. 5.01
g5197	Proteolysis	Neprilysin-like protease	—– vs. 5.06
g3477	Proteolysis	Trypsin-like protease	4.70 vs. —–
g6936	Proteolysis	Peptidase family C14	2.32 vs. —–
g3877	Proteolysis	Trypsin-like protease	6.55 vs. 7.08
g8739	Signaling molecules	C-type lectin	3.51 vs. 1.91

ACC promotes gametophyte maturation, which then releases spores and the individual disappears. By contrast, ACC suppresses the growth of sporophytes, but the individuals are maintained. The different types of proteases induced by ACC might reflect the differences in ACC responsiveness between generations. A comparison of the specific expression patterns and target substrate proteins of ACC-responsive proteases across generations will advance our understanding of the role of proteolysis in mobilizing nitrogen resources during the *Pyropia* life cycle.

g8739, which contains a C-type lectin domain, was upregulated in ACC-treated *P. yezoensis*. C-type lectins were among the first animal lectins discovered (Zelensky and Gready, 2005). In mammals, C-type lectins are secreted molecules or transmembrane proteins. Although there is little information on C-type lectins in higher plants, they are known to be involved in phytohormone signaling—for example, the *Arabidopsis thaliana* lectin ArathEULS3, the mRNA levels of which increase in *Arabidopsis* cell suspension cultures after abscisic acid (ABA) treatment, has been reported to interact with the ABA receptor PYL9, one of the most important players in ABA signaling (Dubiel et al., 2020). In addition to the C-type lectin domain, g8739 contains the von Willebrand A domain, which is a well-studied domain involved in cell adhesion ECM proteins and integrin receptors (Whittaker and Hynes, 2002). Thus, the upregulation of g8739 suggests that the gene is an important player in ACC signaling in *P. yezoensis*.

Taken together, these transcriptomic changes are consistent with our physiological observations that ACC treatment inhibited the growth and induced the degradation of photosynthetic pigments in *P. yezoensis* sporophytes. This suggests that ACC induces transcriptional changes associated with processes similar to plant senescence. However, it should be noted that these conclusions are based on transcriptomic data, and further biochemical analyses are needed to confirm whether these gene expression changes translate into functional metabolic shifts. Moreover, although ethylene is a well-known regulator of senescence in higher plants, the mechanisms by which its precursor ACC affects the sporophytes of *P. yezoensis* may differ due to species-specific factors and the unique biology of red algae.

## Conclusions

Ethylene has long been known as a major hormone hastening leaf and flower senescence in higher plants. To our knowledge, this is the first study suggesting that ACC may have a senescence-like effect in both plants and algae, potentially independent of ethylene. Previous studies revealed that ACC promotes the transition of macroscopic gametophytes to microscopic sporophytes in *P. yezoensis*. In this study, ACC treatment induced catabolic processes and repressed anabolic processes in microscopic sporophytes. *P. yezoensis* generally grows as gametophytes during winter, when nutrients, especially nitrogen, are abundant, and as sporophytes during summer, when nutrient availability is low. Therefore, considering the effects of ACC on both gametophytes and sporophytes, it is possible that ACC contributes to reducing the metabolic activity or nutrient demand of algae under nutrient-limited conditions, potentially serving as a signal to adjust physiological processes according to environmental nutrient availability.

## Data Availability

The datasets presented in this study can be found in online repositories. The names of the repository/repositories and accession number(s) can be found in the article/**Supplementary Material**.
