# Supplementary material for: 1-Aminocyclopropane-1-carboxylic acid induces resource reallocation in Pyropia yezoensis sporophytes
Source: Front Plant Sci. 2025 Aug 15;16:1632530. doi: 10.3389/fpls.2025.1632530 (PMC12394230; doi:10.3389/fpls.2025.1632530)
Supplement: Supplementary file 1 [file Presentation1.pptx]

## Slide 1
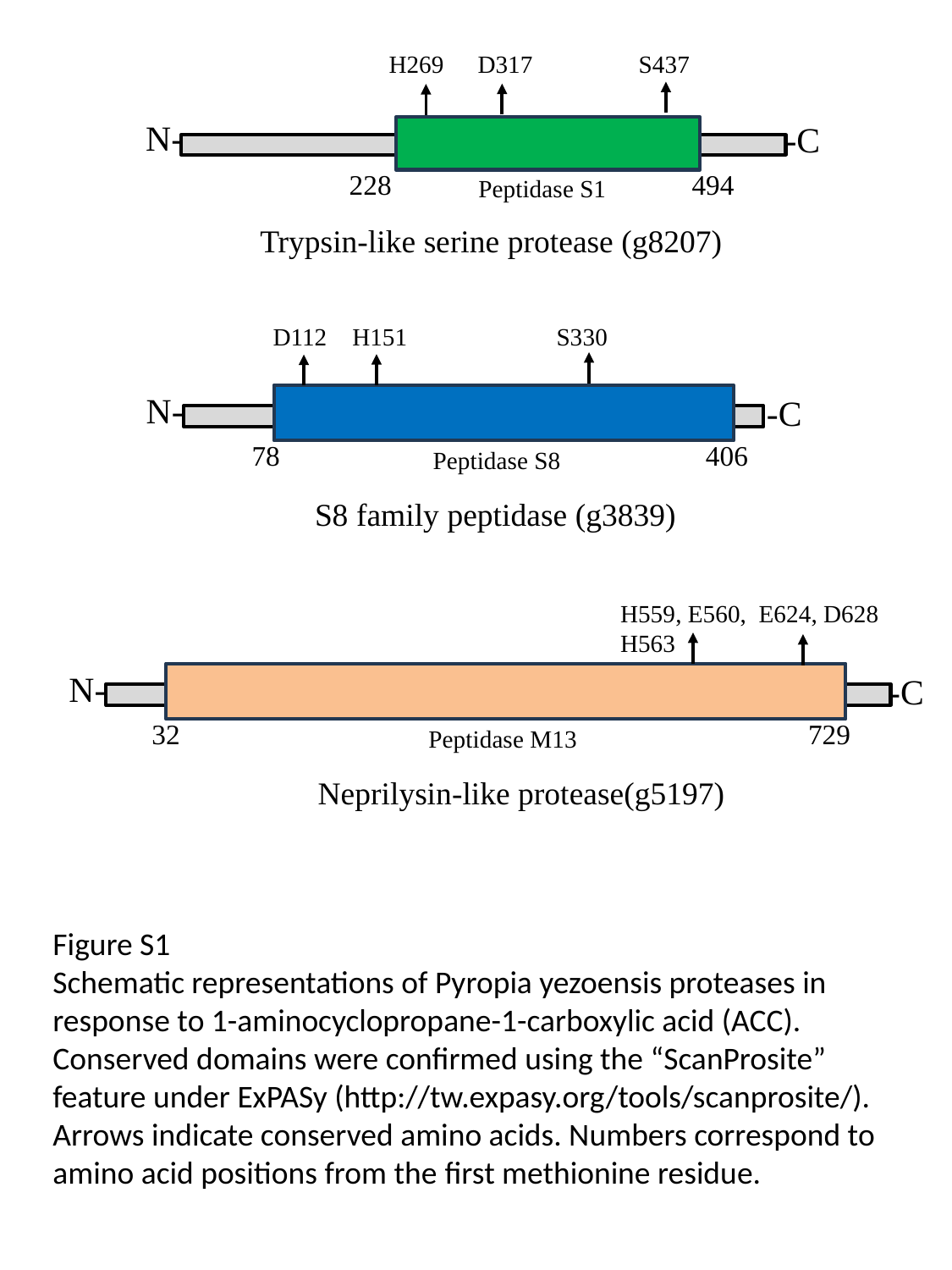

H269
D317
S437
N-
-C
228 494
Peptidase S1
Trypsin-like serine protease (g8207)
D112
H151
S330
N-
-C
78 406
Peptidase S8
S8 family peptidase (g3839)
E624, D628
H559, E560,
H563
N-
-C
32 729
Peptidase M13
Neprilysin-like protease(g5197)
Figure S1
Schematic representations of Pyropia yezoensis proteases in response to 1-aminocyclopropane-1-carboxylic acid (ACC).
Conserved domains were confirmed using the “ScanProsite” feature under ExPASy (http://tw.expasy.org/tools/scanprosite/). Arrows indicate conserved amino acids. Numbers correspond to amino acid positions from the ﬁrst methionine residue.
